# Characterization of designed, synthetically accessible bryostatin analog HIV latency reversing agents

**DOI:** 10.1016/j.virol.2018.05.006

**Published:** 2018-07

**Authors:** Matthew D. Marsden, Xiaomeng Wu, Sara M. Navab, Brian A. Loy, Adam J. Schrier, Brian A. DeChristopher, Akira J. Shimizu, Clayton T. Hardman, Stephen Ho, Christina M. Ramirez, Paul A. Wender, Jerome A. Zack

**Affiliations:** a Department of Medicine, Division of Hematology and Oncology, University of California Los Angeles, Los Angeles, CA 90095, United States; b Departments of Chemistry and of Chemical and Systems Biology, Stanford University, Stanford, CA 94305, United States; c Department of Microbiology, Immunology, and Molecular Genetics, University of California Los Angeles, Los Angeles, CA 90095, United States; d Department of Biostatistics, School of Public Health, University of California Los Angeles, Los Angeles, CA 90095, United States

**Keywords:** HIV, Latency, PKC, Protein kinase C, Reservoir, Cure, Therapy, Bryostatin, Analogs, Bryolog

## Abstract

HIV latency in resting CD4+ T cell represents a key barrier preventing cure of the infection with antiretroviral drugs alone. Latency reversing agents (LRAs) can activate HIV expression in latently infected cells, potentially leading to their elimination through virus-mediated cytopathic effects, host immune responses, and/or therapeutic strategies targeting cells actively expressing virus. We have recently described several structurally simplified analogs of the PKC modulator LRA bryostatin (termed bryologs) designed to improve synthetic accessibility, tolerability in vivo, and efficacy in inducing HIV latency reversal. Here we report the comparative performance of lead bryologs, including their effects in reducing cell surface expression of HIV entry receptors, inducing proinflammatory cytokines, inhibiting short-term HIV replication, and synergizing with histone deacetylase inhibitors to reverse HIV latency. These data provide unique insights into structure-function relationships between A- and B-ring bryolog modifications and activities in primary cells, and suggest that bryologs represent promising leads for preclinical advancement.

## 1 Introduction

Infection with human immunodeficiency virus (HIV) almost invariably causes severe damage to the host immune system, leading to the development of acquired immunodeficiency syndrome (AIDS). This process can be inhibited by the administration of antiretroviral therapy (ART), which blocks HIV replication and often reduces HIV RNA viral loads to levels below the limit of detection using standard clinical assays (approximately 50 copies per mL of plasma) (Dinoso et al., 2009; Gulick et al., 1997; Perelson et al., 1997). However, if ART is stopped, viral replication rapidly resumes allowing disease progression to continue (Chun et al., 1999a). As a result, ART requires strict and life-long compliance to maintain suppression. While multiple factors might contribute to the persistence of HIV during ART, one important source of replication-competent HIV in treated patients is latently-infected CD4 + T cells (Chun et al., 1995; Finzi et al., 1999; Finzi et al., 1997; Wong et al., 1997). These long-lived latently-infected cells harbor integrated proviruses that express little or no viral RNA and no viral proteins, but episodically produce infectious virions upon appropriate stimulation of the host cell (Chun et al., 1997; Finzi et al., 1997). Elimination of these reservoir cells, the chronic source of re-infection, is therefore critical for HIV eradication from infected individuals.

One approach for purging latent reservoir cells is to induce expression of the latent provirus, thereby rendering the host cell susceptible to viral cytopathic effects, immune effector mechanisms, and/or therapeutic approaches targeted towards viral proteins (Reviewed in (Marsden and Zack, 2009, Marsden and Zack, 2010; Marsden and Zack, 2015)). Several clinical efforts to induce HIV expression in latently infected cells have been undertaken, including administration of interleukin 2 (IL-2) either alone (Chun et al., 1999b) or in combination with anti-CD3 antibodies (OKT3) (Kulkosky et al., 2002; Prins et al., 1999; van Praag et al., 2001). Histone deacetylase inhibitors (HDACi) (Archin et al., 2012; Lehrman et al., 2005; Rasmussen et al., 2014) and the anti-alcoholic-abuse agent disulfiram (Elliott et al., 2015) have been tested in a similar manner. More recent work in this area includes a combined approach using Vacc-4 × , recombinant human granulocyte macrophage colony stimulating factor vaccination, and HDACi romidepsin, which was tested in a phase 1B/2A trial (Leth et al., 2016). The treatments described in these important pioneering studies had varying effects on HIV expression and the relative frequency of latently infected cells, with several capable of increasing HIV RNA expression or inducing limited reduction of the latent reservoir in a subset of patients. However, none of them entirely eliminated replication-competent HIV from the infected individuals. The more aggressive therapies involving OKT3 and/or IL-2 also resulted in toxic side-effects due to generalized immune activation (Prins et al., 1999, van Praag et al., 2001). This issue of toxicity associated with HIV LRAs is challenging because expression of HIV proviral DNA is connected to the activation state of the host cell, prompting concerns that the most effective HIV latency reversal agents might also cause global T cell activation, resulting in hypercytokinemia or a “cytokine storm” that could create unacceptable risks and thus preclude their clinical use. Therefore, while the “kick and kill” approach for eliminating latent HIV has been explored in proof-of-concept clinical studies, more effective and better tolerated agents are required if latency purging strategies are to be fully effective.

Protein kinase C (PKC) modulators, including prostratin, ingenol, and bryostatin are of interest in the context of HIV eradication efforts because they can activate the transcription factor NF-kappa B (Williams et al., 2004) and induce HIV from latency in various cell and animal models, as well as primary cells from ART-treated patients (Baxter et al., 2016, Beans et al., 2013, Bullen et al., 2014, Darcis et al., 2015, DeChristopher et al., 2012, Jiang et al., 2015, Kinter et al., 1990, Marsden et al., 2017, Perez et al., 2010, Qatsha et al., 1993). Indeed, the first phase I clinical trial of bryostatin 1 (hereafter referred to as bryostatin) has been conducted in ART-treated, HIV-infected individuals. This study found bryostatin to be safe at the 10 and 20 μg/m^2^ doses tested, with the results indicating that higher doses would likely be needed to induce the desired effects on PKC activity in vivo (Gutierrez et al., 2016). Higher doses (up to approximately 50 μg/m^2^) are tolerated, as demonstrated in several oncology trials with bryostatin, but the identification of analogs with an expanded therapeutic window is a significant objective of ongoing research.

Despite bryostatin's clinical promise, this lead compound is isolated in very low and variable yields from its marine source (14 t of *Bugula neritina* yielded only 18 g of Good Manufacturing Practice-grade [GMP] bryostatin), raising cost and environmental concerns about its sustainable supply from natural sources (Schaufelberger et al., 1991). To address this problem, aquaculture was tried but abandoned (Mendola, 2003). Synthetic biological approaches remain in early stages due to complications arising from cultivation of the symbiotic bacterium that produces bryostatin (Miller et al., 2016, Trindade-Silva et al., 2010). The current supply of GMP bryostatin produced in the 1990s is nearly depleted. Thus while bryostatin continues to serve as a significant therapeutic lead and an important clinical candidate for multiple indications, the aforementioned therapeutic window and supply issues have hampered its advancement. Recognizing that bryostatin, like many natural products, is neither evolved nor optimized for the treatment of human disease, we previously reported the first analogs of bryostatin that are more synthetically accessible and exhibit activities comparable or better than bryostatin (Wender et al., 2014, Wender et al., 2015). More recently we reported a scalable total synthesis of bryostatin that addresses the clinical supply problem, and importantly is readily adapted to enable the design and synthesis of superior analog compounds, lead examples of which are evaluated herein (Wender et al., 2017).

**Fig. 1:**
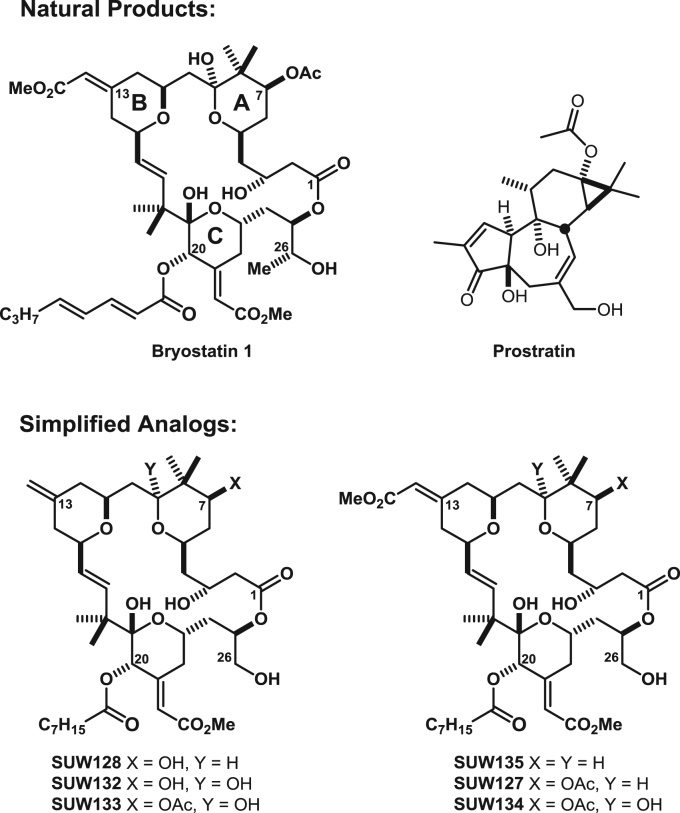
Structures of compounds. Chemical structures of bryostatin 1, prostratin, and simplified bryostatin analogs (bryologs) are shown. Analogs SUW128, SUW133, SUW135, SUW127, and SUW134 here correspond to previously published (DeChristopher et al., 2012) analogs 2, 4, 5, 6, and 7 respectively. SUW132 corresponds to compound 4.55 (Schrier, 2011), and was prepared in an analogous manner to SUW128 and SUW133 (Wender et al., 2014).

To address these issues of supply and sub-optimal activity, we previously reported a function-oriented approach to designed synthetically-accessible bryostatin analogs (DeChristopher et al., 2012, Wender et al., 1988). We demonstrated that we could effectively recapitulate the PKC affinities and activities of the natural product with bryostatin analogs or “bryologs” featuring structural variations in the A- and B-rings of the macrocyclic scaffold (Fig. 1). This approach also provides the opportunity to tune the activity of analog compounds to optimize their performance via targeted structural manipulation of the bryostatin scaffold. Significantly, these designed bryologs potently induce HIV expression in a J-Lat model for HIV latency *(*DeChristopher et al., 2012*)*. We further established that one particularly efficacious bryolog (SUW133) could induce HIV from latency ex vivo from CD4 + T cells derived from HIV-infected ART-treated patients and in vivo in humanized BLT mice (Marsden et al., 2017).

In the current study, we address the activities of these bryologs as required for their preclinical advancement with a focus on their comparative ability to alter HIV entry receptor levels and induce proinflammatory cytokines in primary peripheral blood mononuclear cells (PBMC), affect HIV spread in activated CD4 + T cells, and synergize with HDACi to reverse HIV latency. These experiments provide important insights into how structural variations of bryologs affect HIV latency reactivation and influence subsequent viral spread. Significantly, we have found that improved HIV latency reversal activity can be achieved with lead bryologs without a corresponding increase in induction of potentially damaging cytokines.

## 2 Results

### 2.1 Bryologs reduce cell surface HIV entry receptor levels

**Fig. 2:**
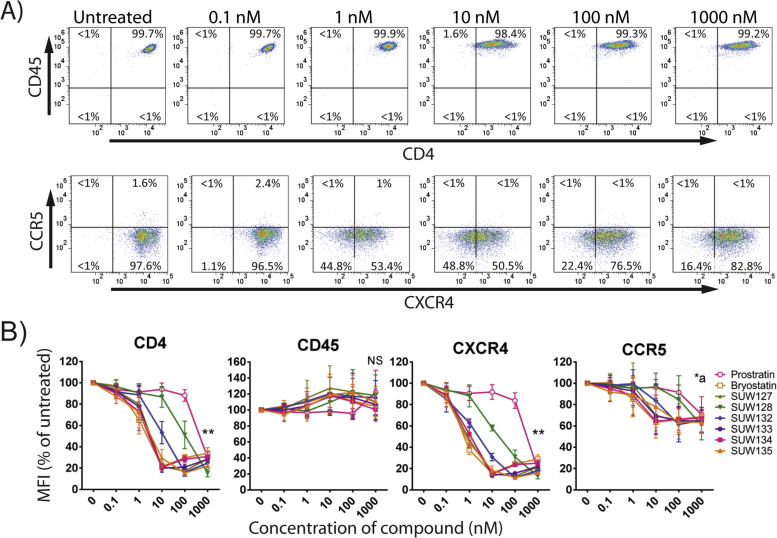
Compound-induced reduction of cell surface molecules in primary CD4 + T cells. A) CD4 + T cells from a healthy human donor were exposed to the indicated concentrations of bryostatin for 24 h before harvesting. Expression levels of cell surface CD45, CD4, CCR5, and CXCR4 were assessed by flow cytometry. Representative flow cytometry plots are shown. B) Cells were treated as described in part A with the indicated compounds. The mean fluorescent intensity (MFI) is provided as a percentage of that obtained for untreated cells from the same donor. ** all compounds induced significant reductions (p < 0.001) in receptor levels (untreated vs 1000 nM). NS no compound induced significant reductions in receptor levels (untreated vs 1000 nM). *a p < 0.05 (untreated vs. 1000 nM) for all compounds except prostratin, SUW128, and SUW132 using a 2-sided *t*-test. Error bars = SEM, N = 3 different primary cell donors.

Structures for the synthetic bryologs, bryostatin and prostatin used in this study are provided (Fig. 1). We first explored whether the novel bryologs share potentially beneficial activities with the parent compound bryostatin, beyond their ability to induce HIV from latency. Not unlike bryostatin and prostratin (a non-tumor promoting phorbol ester that similarly activates latent HIV by activating protein kinase C), the bryologs are pan-PKC modulators, binding to all novel and conventional PKC isoforms, although prostratin exhibits 10–100-fold lower affinities. One property of both bryostatin and prostratin is the capacity to transiently reduce cell surface levels of the receptors CD4, CCR5, and CXCR4 (Kulkosky et al., 2001, Mehla et al., 2010, Rullas et al., 2004) through internalization and degradation in a PKC-dependent pathway (Hezareh et al., 2004). Cell surface CD4 expression is required for the entry of nearly all primary HIV isolates (Dalgleish et al., 1984), and CCR5 (Alkhatib et al., 1996, Deng et al., 1996, Dragic et al., 1996) or CXCR4 (Feng et al., 1996) are essential coreceptors for infection with R5-tropic or X4-tropic HIV isolates, respectively. We previously demonstrated that a panel of PKC modulators (including several compounds shown in Fig. 1) induced expression of CD69 at the same concentrations as that required to induce HIV from latency (Marsden et al., 2017), however their effects on HIV receptor expression have not been defined. Therefore, primary CD4 + T cells were isolated from healthy donors and stimulated for 24 h with different concentrations of prostratin, bryostatin, or one of several bryologs. Expression levels of CD4, CCR5, CXCR4, and CD45 were then quantified by flow cytometry (Fig. 2). CD45 is expressed on all leukocytes and was included for control and comparison purposes because its surface expression levels have not been reported to be affected by PKC modulation. In line with their relative PKC affinities, prostratin significantly (p < 0.001) downregulated the expression of CD4 and CXCR4 at a concentration of 1 µM, whereas bryostatin downregulated the expression of these receptors and also CCR5 at concentrations as low as 1 nM (Figs. 2A and 2B). Similarly, the bryologs tested also downregulated these receptors at low nanomolar concentrations. The C7-acetoxy or C7-deoxy analogs (SUW133, SUW135, SUW127, and SUW134) are more potent than the C7-hydroxyl analogs (SUW128 and SUW132), potentially in agreement with recent molecular dynamics studies showing that ligands bound to PKC could influence its positioning in a membrane microenvironment and thus function (Ryckbosch et al., 2017). CD45 was not significantly downregulated by any compound tested. Overall, the higher concentrations of compound induced greater than 80% reductions in CD4 and CXCR4 mean fluorescent intensity (MFI). The majority of CD4 + T cells obtained directly from healthy human donors are not activated, and CCR5 expression levels are typically low in these resting cells. However, each of the tested compounds except for prostratin, SUW128, and SUW132 induced a significant (p < 0.05) but modest decline in CCR5 MFI (Fig. 2B).

### 2.2 Effect of bryologs on short-term HIV spread (replication in culture)

**Fig. 3:**
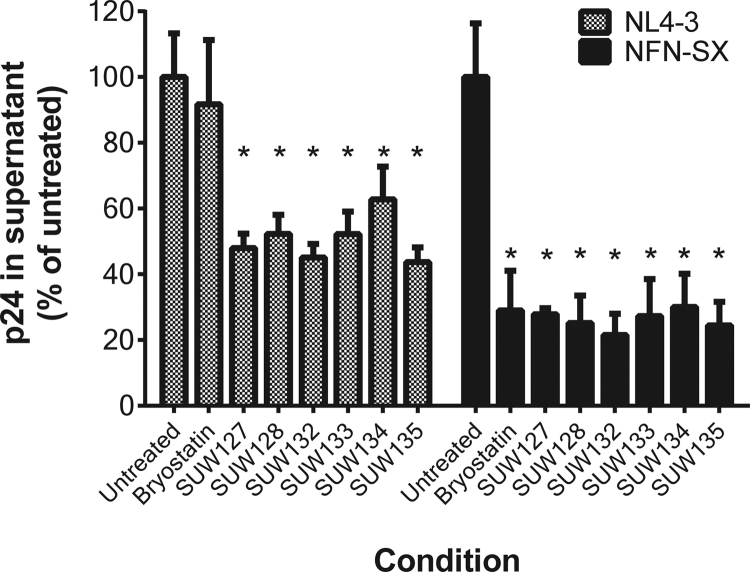
Short-term HIV spread in costimulated CD4 + T cells treated with compounds. Costimulated CD4 + T cells were infected with an X4 (NL4-3) or R5 (NFN-SX) strain of HIV and then treated with 100 nM of bryostatin 1 or the indicated analog. Culture supernatants were harvested at d3 post-infection and p24 concentrations were quantified by ELISA. Average p24 concentrations in untreated cultures were 77.4 ng/mL for NL4-3 and 37.1 ng/mL for NFN-SX. Error bars represent ± 1 Standard Error (N = 4 different primary cell donors). * Indicates p < 0.05 compared with untreated control using a 2-sided *t*-test.

The net effect of treatment with PKC activating agents such as bryostatin and prostratin on HIV spread is the result of a complex set of interactions influenced by the positive and negative effects of these compounds on the different stages of the HIV life cycle. For example, prostratin and bryostatin can each downregulate HIV entry receptor levels and inhibit virus entry (Fig. 2), but upregulate HIV expression from integrated proviral DNA (DeChristopher et al., 2012, Kulkosky et al., 2001). To determine the effects of the novel bryologs on HIV spread in culture, primary CD4 + T cells were costimulated via CD3 and CD28 ligation and then infected with either the CXCR4-tropic HIV strain NL4-3 or the CCR5-tropic HIV strain NFN-SX. This latter virus is identical to NL4-3 except for the *Stu*I-*Xho*I region (encompassing most of the virus envelope gene), which is derived from the genome of the primary CCR5-tropic HIV isolate JR-FL (O'Brien et al., 1990). Immediately following the initial 2 h infection period the cells were placed in cultures containing a 100 nM concentration of bryostatin or individual bryologs. HIV spread under these different conditions was measured after 3 days by quantifying HIV p24 (viral capsid) protein levels in the culture supernatants. Each of the tested bryologs significantly (p < 0.05) inhibited spread of both HIV variants in this assay (Fig. 3). Interestingly, during NL4-3 infection bryostatin induced a smaller non-statistically significant trend for reducing spread, likely reflecting small differences in the balance between enhancement versus inhibition of individual HIV life cycle stages between bryostatin and bryologs mentioned above. Standard MTT cytotoxicity assays conducted using CEM T cells or primary human PBMCs demonstrated that the indicated concentrations of compounds were not cytotoxic themselves (Fig S1and S2). In addition, flow cytometric forward and side scatter profiles showed no evidence of cytotoxicity under these conditions.

### 2.3 Cytokine induction in primary peripheral blood mononuclear cells

**Fig. 4:**
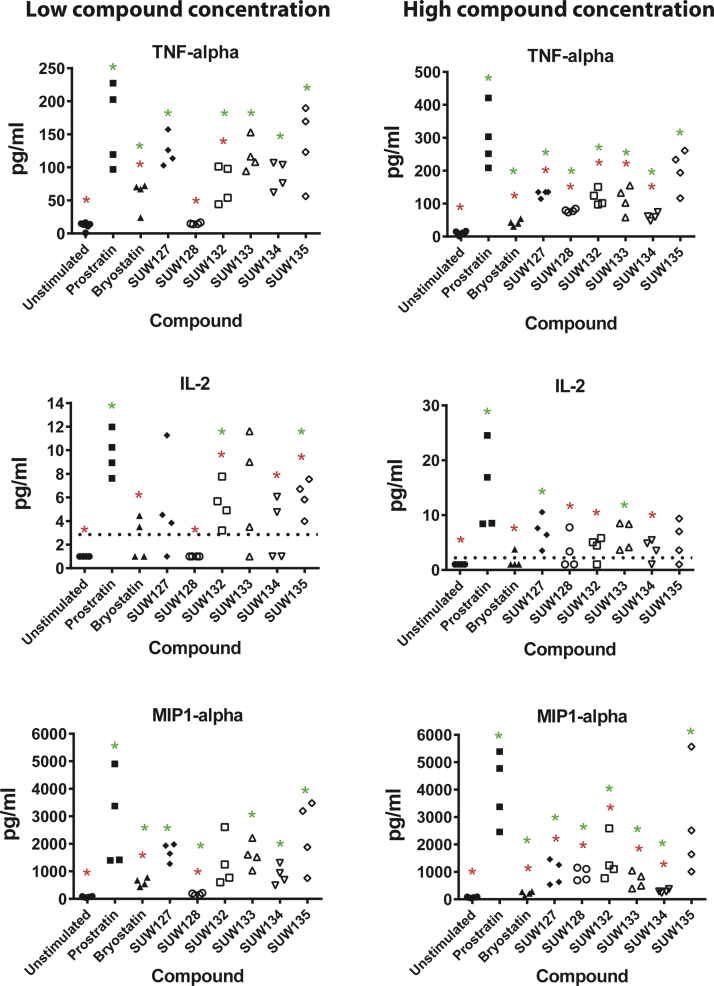
Cytokine induction by compounds in primary PBMC cultures. Primary human PBMCs were exposed to the indicated compound for 18 h and then cytokine concentrations in the culture supernatant were measured using a multiplex bead assay. Results for tumor necrosis alpha (TNF-alpha), interleukin-2 (IL-2), and macrophage inflammatory protein 1 alpha (MIP1-alpha) are shown. Compound concentrations were based on a “low concentration” of prostratin (1 μM) or bryostatin 1 (10 nM) that induces latent HIV expression in approximately 20% J-Lat 10.6 cells. A low concentration of 10 nM was also used for the bryologs. The “High concentration” corresponds to 10 μM prostratin and 100 nM of bryostatin 1 or the bryologs. Broken lines in IL-2 charts indicate the lower level of detection for the assay. Results from two primary cell donors (2–3 individual stimulations for each donor) are presented. Red * indicates p < 0.05 compared with prostratin using a 2-sided *t*-test. Green * indicates p < 0.05 compared with unstimulated control using a 2-sided *t*-test.

To assess whether bryologs induce cytokine production in PBMCs, and to compare this capacity with that of the natural compounds prostratin and bryostatin, we treated primary human PBMCs from healthy donors with compounds for 18 h then measured the concentration of several inflammation-related cytokines in the culture supernatants. The amount of prostratin and bryostatin used for stimulation was based on the concentration required to activate HIV from latency in approximately 20% of J-Lat 10.6 cells. This corresponded to concentrations of 1 µM prostratin and 10 nM bryostatin. For consistency with bryostatin, the bryologs were also tested at 10 nM concentrations. Additionally, 10-fold higher compound concentrations were tested in a similar manner (Fig. 4).

Levels of TNF-alpha, IL-2 and MIP1-alpha that were induced in the PBMC supernatant are shown (Fig. 4). TNF-alpha is particularly important in this context because it is extremely toxic when present at high levels in vivo. IL-2 can support proliferation of activated T cells and might therefore exacerbate the immunological side-effects of latency activation by these compounds. MIP-1 alpha is a ligand for CCR5 and is thus of interest because it can inhibit replication of R5-tropic HIV isolates. Overall, the bryologs induced lower levels of cytokine production than did prostratin, but the magnitude of cytokine production varied depending on analyte compound structure, suggesting that latency activation and cytokine induction are controlled differently by different agents, and thus could each be controlled by structural variations to the bryostatin scaffold.

**Fig. 5:**
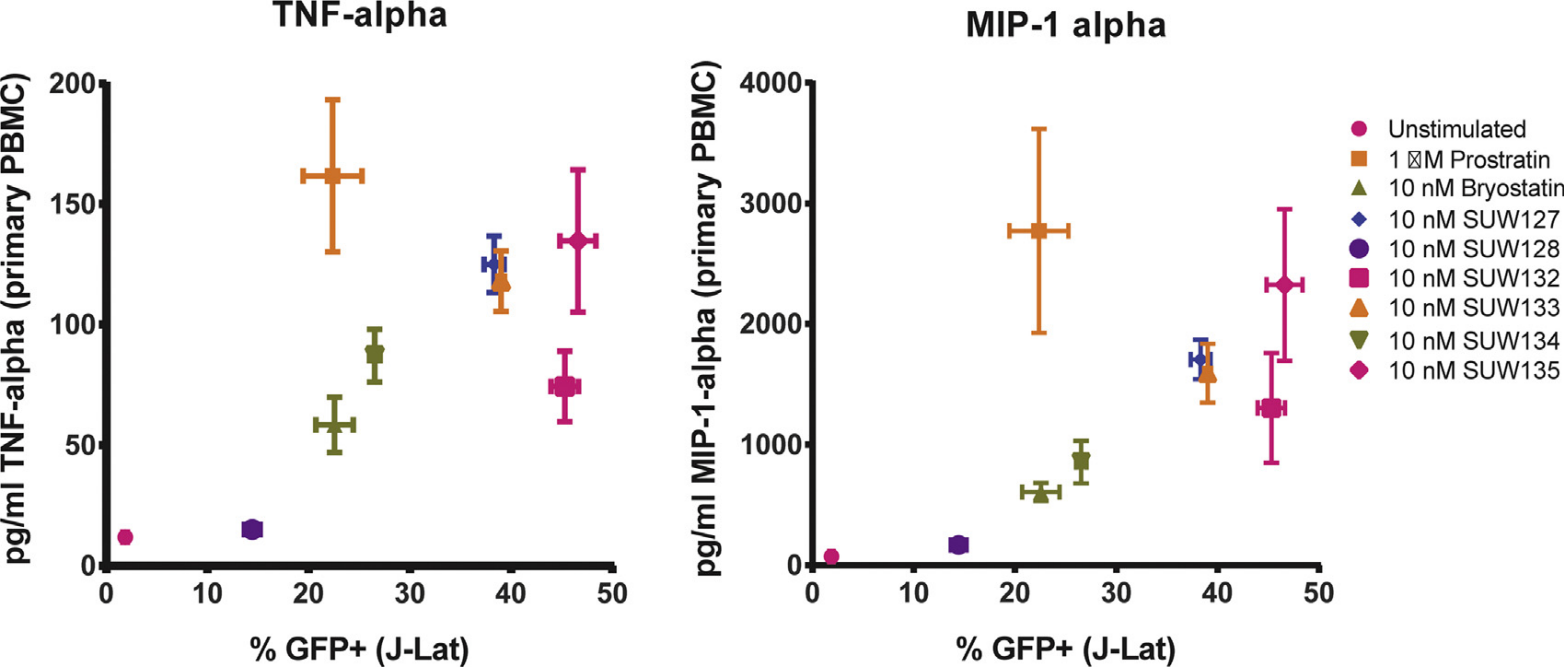
Comparison between HIV latency activation and cytokine induction. The amount of TNF-alpha (left panel) or MIP-1 alpha (right panel) produced in primary PBMC cultures (from Fig. 4) is plotted against the percentage of cells activated with the same concentration of compound in the J-Lat 10.6 latency assay. GFP+ cells in the J-Lat assay represent those cells that have been activated from latency during a 24 h incubation. Relevant statistical comparisons using a 2-sided *t*-test are described in the main text. Error bars represent ± 1 Standard Error.

To more easily compare the capacity of the compounds to activate HIV from latency with their induction of cytokines in PBMCs, these data were directly compared for two relevant cytokines. The percentage of J-Lat 10.6 cells demonstrating activation of the latent provirus following a 48 h stimulation with each compound was plotted against cytokine production shown in Fig. 4 for TNF-alpha and MIP-1 alpha (Fig. 5). At the concentrations indicated, several bryologs induced HIV from latency more effectively with either no increase, or only modest increases in cytokine production compared with bryostatin. For example, analog SUW132 induced approximately twice as many latently-infected J-Lat cells to express HIV (p < 0.0001) while not significantly increasing TNF-alpha or MIP-1 alpha production (p > 0.1) in PBMCs (Fig. 5). The majority of the bryologs also showed reduced cytokine production at concentrations that induce similar or greater levels of latency activation than prostratin. Hence, this decoupling of desired and undesired effects suggests that these or related bryologs might prove advantageous over the natural product by both improving latency activation and reducing toxicities associated with induction of inflammatory cytokines.

### 2.4 Synergistic activation of HIV from latency when used with HDACi

**Fig. 6:**
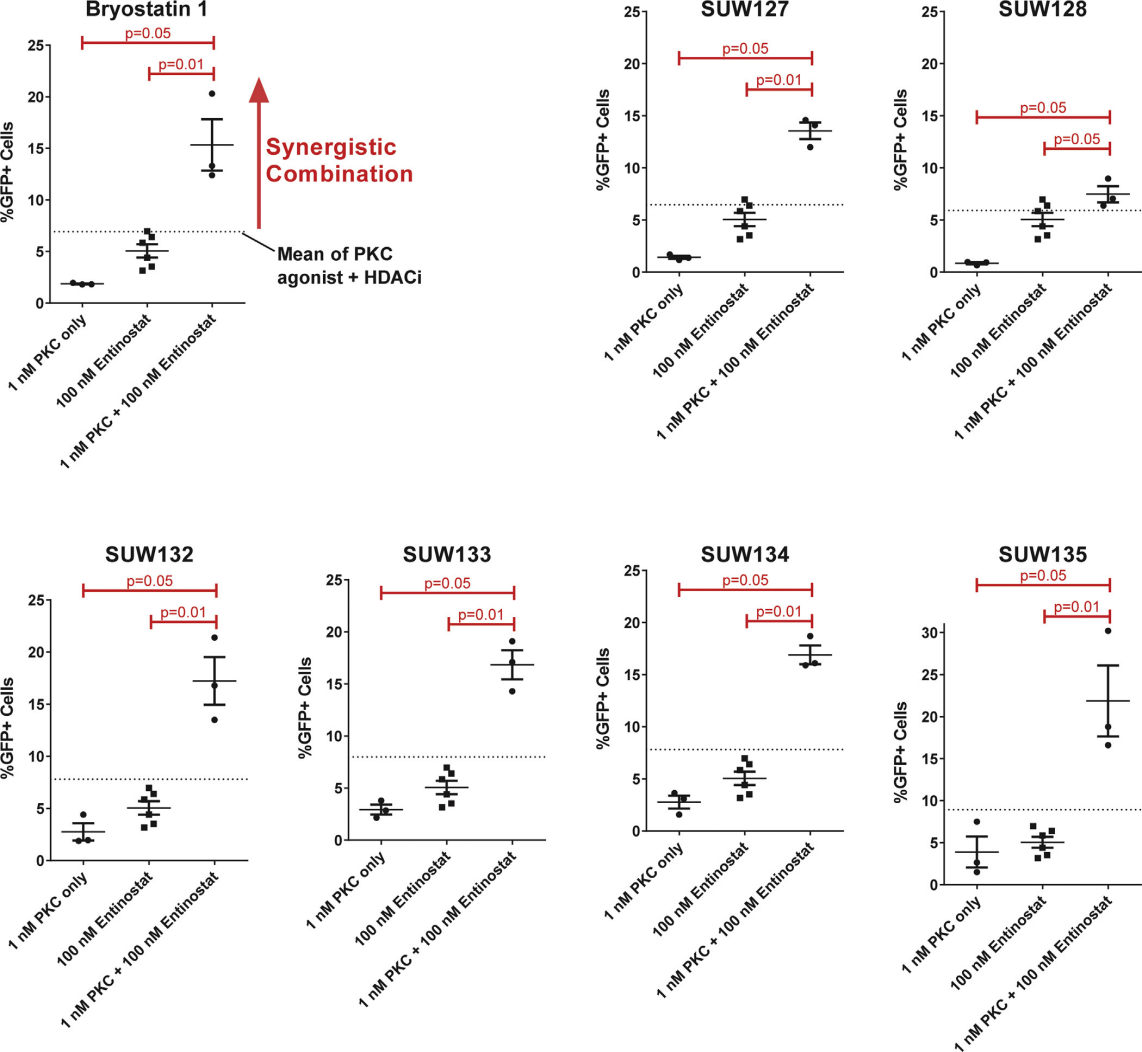
Synergistic reactivation of HIV from latency. J-Lat 10.6 cells were exposed to sub-optimal concentrations of the indicated PKC modulator alone (bryostatin 1 or SUW-designated compounds), the HDACi entinostat alone, or a combination of the two for 48 h. Lines representing the sum of values for PKC modulator alone and entinostat alone (i.e. additive an additive response) are shown on each plot. The same entinostat alone values are presented in each plot to facilitate comparison. Data are from 3 to 6 independent experiments. Groups were compared using an exact independent one-sided Wilcoxon rank sum test. P-values less than 0.05 are considered significant.

An important direction of research in this area with potential clinical consequences is the use of combinations of agents to enhance activation at lower concentrations while also lowering off target effects. As such, combinations of agents operating through different pathways could prove more effective than either agent alone through synergistic effects. For example, combinations consisting of a PKC activating agent and a histone deacetylase inhibitor (HDACi) have shown synergistic HIV latency activation in some cases (Albert et al., 2017, Perez et al., 2010, Zaikos et al., 2018). To explore whether the current set of bryologs can also synergize with well-studied HDACi's we selected sub-optimal concentrations of each PKC modulator and one of three different HDACi (entinostat, vorinostat, and panobinostat) to test individually or in combination for their ability to reverse HIV latency in J-Lat 10.6 cells. We found that bryostatin and each bryolog synergized in combination with entinostat (Fig. 6), vorinostat (Fig S3) and panobinostat (Fig S4) to produce a greater that additive induction of HIV from latency. This suggests that such combinations are feasible with bryologs, and thus could be used to further enhance efficacy and tolerability over bryostatin (Marsden et al., 2017). By enhancing activation while requiring lower dosing of compound, such synergistic effects could also reduce off-target effects.

### 2.5 PKC binding evaluations

**Fig. 7:**
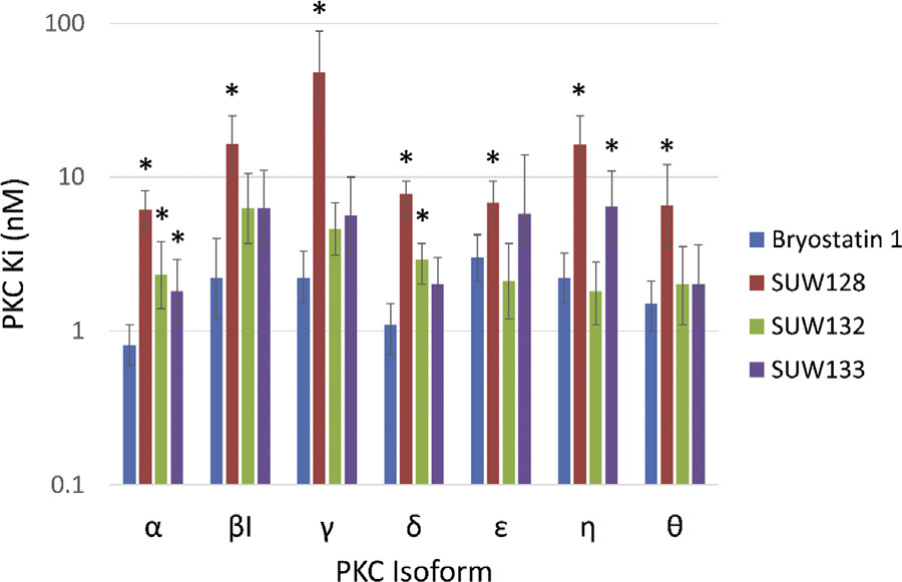
Binding affinities to conventional and novel PKC isoforms. Lower Ki (nM) values indicate more effective binding. *= p < 0.05 using a 2-sided *t*-test.

We have previously published PKC isoform binding affinities of bryostatin and SUW133 (Marsden et al., 2017). For completeness in understanding how structural variations in bryologs influence PKC binding and downstream effects on HIV, here we have quantified binding affinities for two additional bryologs against the full panel of conventional and novel PKC isoforms. It is noteworthy that these and previously reported bryologs are pan-PKC binders, although SUW128 is a somewhat less good binder and less potent than SUW132 and SUW133 (Table 1 and Fig. 7). This suggests that in the absence of the C13 enoate carbonyl and in the presence of a polar C7 functionality, C9 oxygenation is necessary for strong ligand binding to PKC. This could explain SUW128's decreased potency in HIV entry receptor downregulation (Fig. 2) and HIV latency reversal (Fig. 5) compared with SUW132 and SUW133. Significantly, and of potential therapeutic consequence, because not all PKC isoforms are implicated in activation associated with LRA, the differing isoform affinities of SUW128 versus, for example SUW132, suggests that variations in structure could eventually lead to isoform selective agents.

**Table 1:** PKC binding affinities for select compounds. Values for bryostatin 1 and SUW133 are from Marsden et al. (2017). Lower Ki (nM) values indicate more effective binding. 95% confidence intervals are in parentheses.

	**PKC Ki (nM)**						
	**α**	**βI**	**γ**	**δ**	**ε**	**η**	**θ**
**Bryostatin 1**	0.81 (0.6–1.1)	2.2 (1.2–4.0)	2.2 (1.5–3.3)	1.1 (0.7–1.5)	3.0 (2.1–4.2)	2.2 (1.5–3.2)	1.5 (1.0–2.1)
**SUW128**	6.1 (4.6–8.1)	16.4 (11–25)	48 (26–89)	7.8 (5.5–11)	6.8 (4.9–9.4)	16.3 (11–25)	6.5 (3.5–12)
**SUW132**	2.3 (1.4–3.8)	6.3 (3.7–10.5)	4.6 (3.1–6.8)	2.9 (2.0–4.0)	2.1 (1.2–3.7)	1.8 (1.1–2.8)	2.0 (1.1–3.5)
**SUW133**	1.8 (1.1–2.9)	6.3 (3.5–11)	5.6 (3.1–10)	2.0 (1.3–3.0)	5.8 (2.4–14)	6.4 (3.6–10.9)	2.0 (1.1–3.6)

## 3 Discussion

HIV latency within resting memory CD4 + T cells provides a mechanism for long-lived persistence of the virus in ART-treated patients (Finzi et al., 1999), thus requiring lifelong ART treatment and associated cost, compliance and chemoexposure issues. Reduction or elimination of the latent reservoir is the key to interruption of therapy or eradication, respectively. Consequently, compounds like prostratin and bryostatin that can induce HIV from latency via PKC activation are of research and clinical interest in HIV eradication efforts. Indeed, the former has been advanced preclinically and the latter has been entered in a phase I clinical trial (Gutierrez et al., 2016). However, the previous limited availability of these agents has hampered efforts to identify more effective and better tolerated candidates (Barr et al., 2009, Bullen et al., 2014). We previously addressed the supply of prostratin and bryostatin (Wender et al., 2017, Wender et al., 2008), opening access to their analogs as described previously and advanced herein. Our previous study of earlier bryologs using a cell line HIV latency model (DeChristopher et al., 2012) provided encouraging results on this approach to eradication. Subsequently, one particularly potent bryolog termed SUW133 was further tested *ex vivo* in ART-treated patient-derived cells and *in vivo* in a humanized mouse model of HIV latency (Marsden et al., 2017), the outcome of which provided further support for this approach and its clinical potential. The studies described here were designed to build on these earlier findings by investigating additional and hitherto unexplored characteristics of bryologs that are particularly relevant to their preclinical advancement in the context of HIV eradication.

Prostratin and bryostatin can each reduce HIV entry receptor levels (Kulkosky et al., 2001, Mehla et al., 2010, Rullas et al., 2004). This would be advantageous in the context of activation/elimination approaches to purge HIV latency because it could lead to a reduction in HIV spread from the recently activated latently-infected cells, thereby aiding the antiretroviral regimen in containing the activator-induced burst of viral expression. This could further benefit if needed from boosting ART before LRA treatment analogous to pre-exposure prophylaxis treatment. The novel bryologs also caused downregulation of CD4, CXCR4 and CCR5 levels in CD4 + T cells from HIV seronegative donors (Fig. 2). CD45 levels were little affected in these assays. This receptor modulation will be important to take into consideration in future in vivo studies using these agents, where (for example) isolation or analysis of CD4 + T cells or other immune cell subsets might be complicated by the observed transient reductions in cell surface receptor expression (Fig. 2).

Replication of both X4 and R5 variants of HIV in primary CD4 + T cells was also inhibited by treatment with the bryologs (Fig. 3) in a short-term 3 day spreading infection assay, further supporting the possibility that the same PKC activating agents that induce HIV from latency could also help contain the spread of that virus to uninfected cells. The fact that bryostatin can access sites that are poorly penetrated by some antiretroviral drugs such as the central nervous system (Sun and Alkon, 2006, Zhang et al., 1996) make this capacity of bryologs to reduce HIV spread particularly interesting in the context of HIV eradication efforts. Further studies on the enhancement of permeation and/or targeting of these agents, for example, through the use of guanidinium-rich transporters (Stanzl et al., 2013) or lipid/vault nanoparticles (Buehler et al., 2014, Kovochich et al., 2011) could improve the efficacy of this approach.

A key concern with HIV eradication efforts is the damaging effects of global immune cell activation that might be caused as a by-product of the HIV latency induction treatment. Production of pro-inflammatory cytokines is perhaps the most direct and potentially problematic manifestation of this concern. For example, TNF-alpha plays a key role in the development of systemic inflammatory response syndrome and can be lethal when produced at high levels (Tracey and Cerami, 1993). Both prostratin and bryostatin can induce TNF-alpha production in primary cells (Bosco et al., 1997, Kulkosky et al., 2001), and bryostatin treatment has been shown to be capable of inducing some TNF-alpha production following administration to humans (Philip et al., 1993). It is possible that a small amount of inflammation could be beneficial in HIV latency purging strategies, either by inducing more CD4 + T cell activation and thus higher levels of latency induction, or by enhancing immune clearance of the cells expressing viral proteins. Agents that induce high levels of inflammatory cytokines could potentially be used if their toxic side-effects are managed with anti-inflammatory drugs. However, this would add another level of complexity and uncertainty to purging strategies. The more direct approach is to instead identify compounds that activate HIV from latency but induce minimal levels of cytokine production.

To gain a better understanding of the capacity for the novel bryologs to induce cytokines, we treated PBMCs from HIV seronegative donors with prostratin, bryostatin, or bryologs and then quantified the concentration of several cytokines in the culture supernatants. In these assays, prostratin generally induced a higher level of cytokine production than did bryostatin. The bryologs varied in their induction of cytokines in these cultures, but in most cases induced lower levels of cytokine production than did prostratin, thus reflecting the ability of structural modifications in the bryostatin scaffold to effectively tune analog activity (Fig. 4). When the cytokine induction was directly compared with activation of HIV from latency, several bryologs showed favorable profiles, in that they induced more latently-infected cells to express HIV without a concomitant large increase in cytokines such as TNF-alpha or MIP-1 alpha (Fig. 5), further indicating that variations in structure can be used to decouple the two activities. The net effect of a complex cytokine milieu is difficult to predict and likely depends on the *in vivo* context. However, these assays do provide a broad picture of the capacity of different compounds to perturb cytokine profiles in relevant primary cells.

Our finding that each of these bryologs synergize with three different HDACi (Fig. 6, S3, and S4) is consistent with the recent observation that SUW133 can synergize with largazoles (Albert et al., 2017) and suggests that synergy with HDACi's could be a common feature of PKC modulators based on bryostatin scaffolds. This type of combinatorial approach might allow lower doses of both bryologs and HDACi to be used in vivo than would otherwise be necessary to induce latency reactivation, thus further improving in vivo efficacy and tolerability compared with a single-agent approach.

The current study also provides key insights into how structural variations in PKC modulators can affect their biological properties. Previous computational studies have established the importance of binding interactions between the C13 enoate and C9 functionality in the active ligand-PKC-membrane complex (Ryckbosch et al., 2017). The significance of these *in silico* predictions are reflected in the relative PKC binding affinities of SUW132 and SUW133 as compared to SUW128 (Table 1 and Fig. 7). While analogs SUW132 and SUW133 outperform bryostatin-1 at inducing expression of latent proviral DNA in J-lat cells, SUW128 is ineffective (Fig. 5) thus providing much needed information for further analog design. This difference in function can be attributed to their relative affinities for PKC, the upstream effector protein responsible for inducing NF-κB-mediated expression of latent proviral DNA (Table 1 and Fig. 7). In molecular dynamics simulations of the bryostatin-PKC-membrane ternary complex, C7 appears to be imbedded in the hydrophobic interior of the membrane while the C9 hydroxy group establishes a hydrogen bonding network with water molecules at the membrane-cytosol interface (Ryckbosch et al., 2017). As such, we would predict that analogs with a hydrophilic free hydroxy group at C7, such as SUW128 and SUW132, could be less active or behave differently with respect to the bryostatin-PKC-membrane complex. Our data indicate that in analogs with hydrophilic substituents at C7, C9-oxygenation is necessary for strong analog binding. This is consistent with the decreased activity observed with SUW128 in nearly every assay and the superior performance of SUW132 and SUW133. Furthermore, different behavior of the active enzyme complex, mediated by differential substitution in the A- and B-rings of the bryostatin scaffold, could explain the reduced TNF-alpha response induced by the bryologs in primary PBMC cultures relative to other similarly high performing compounds (Fig. 5).

Bryostatin has been in numerous oncology trials and was recently entered into an HIV eradication trial. Prostratin is the active constituent in a traditional medicine used in Samoa and has been advanced preclinically as an LRA. In addition to tolerability, both compounds have shown encouraging in vivo activities that could ultimately be of clinical significance. Both natural products can now be supplied through synthesis (Wender et al., 2017, Wender et al., 2008). Significantly, while the synthetic version of these natural products might be effective clinically, these syntheses now open the door for the design and evaluation of potentially more effective, better tolerated and more accessible analogs. This current study demonstrates that bryologs can downregulate HIV entry receptors, inhibit HIV spread, and induce HIV from latency at concentrations that induce relatively low levels of proinflammatory cytokine production. Furthermore, we show that each of these bryologs can synergize with HDACi, which may represent a useful combinatorial approach in future in vivo studies. Significantly, while the vast body of research on LRAs over the past 20 years has been focused on several natural products, these studies show that more accessible, effective and better tolerated agents can be achieved through synthesis-informed design, thus providing a potentially more effective approach to HIV eradication and leads for other therapeutic indications.

## 4 Materials and methods

### 4.1 Cell isolation and stimulation procedures

Primary cells were cultured in “RF10 medium” consisting of RPMI 1640 medium supplemented with 10% fetal bovine serum (FBS, Omega Scientific), 100 U/mL of penicillin, and 100 μg/mL of streptomycin (Invitrogen). PBMCs from healthy donors were isolated using Ficoll-Paque Plus separation (GE Healthcare). Primary CD4 + T cells were separated from PBMCs by negative immunomagnetic selection using the CD4 + T cell Isolation Kit (Miltenyi Biotec) according to the manufacturer's instructions. Cells were then exposed to compound for 24 h before staining and flow cytometric analysis of receptor levels. Prior to infections cells were costimulated through CD3 and CD28 stimulation to allow HIV infection. During costimulation, cells were activated using plate-bound anti-CD3 (1 μg/mL) and soluble anti-CD28 (100 ng/mL) antibodies in the presence of 20 U/mL of IL-2, using procedures described previously (Marsden et al., 2012). Compound incubations with both freshly isolated (unstimulated) and costimulated cells were performed by seeding 10^5^ cells/well in 100 μL of RF10 media containing the appropriate concentration of compound in wells of a v-bottomed 96-well plate. Unstimulated cells were maintained throughout the compound stimulations in RF10 medium without IL-2. Bryostatin 1 (Tocris bioscience) and Prostratin (LC Laboratories) were obtained commercially, and bryologs were synthesized as previously described (DeChristopher et al., 2012).

### 4.2 Flow cytometry

Samples of 10^5^ cells were suspended in 50 μL of a 1:1 dilution of phosphate buffered saline (PBS):Human AB serum (Sigma). The following fluorescent conjugated antibodies were then added: CCR5 (CD195)-Phycoerythrin (PE, BD Biosciences); CD4-PhycoerythrinTexas Red (ECD, eBioscience); CXCR4 (CD183)-Allophycocyanin (APC, eBioscience); and CD45-Phycoerythrin Cyanin 7 (PC7, BD Biosciences). During staining, cells were incubated at 4 °C for 20 min, washed with PBS, and then resuspended in 2% paraformaldehyde. Stained samples were stored at 4 °C, then samples were run using a Cytomics FC 500 flow cytometer (Beckman Coulter). Data was analyzed using FlowJo (v7) software. Forward and side scatter profiles were used to exclude dead cells.

### 4.3 Virus infections

HIV NL4-3 (Adachi et al., 1986) and NFN-SX (O'Brien et al., 1990) viruses were produced by transient transfection of plasmids into 293FT cells using Lipofectamine 2000 reagent (Invitrogen). For virus production, a total of 6.6 × 10^6^ 293FT cells per dish were seeded in 10-cm diameter tissue culture dishes in 10 mL of Opti-MEM I medium (Invitrogen) containing 10% FBS. The following day, the cells were transfected with 10 μg of plasmid DNA per plate according to the Lipofectamine manufacturer's recommendations. The Lipofectamine/DNA complexes were incubated with cells for 20 h, then the media was replaced with Dulbecco's Modified Eagle Medium (Life Technologies) containing 10% FBS. At day 2 post-transfection, the virus-containing supernatants were harvested and filtered (0.45 µm), and then aliquots were stored at − 80 °C. Viral p24 levels were quantified using an HIV p24 enzyme-linked immunosorbent assay (ELISA) kit (Beckman Coulter).

During infections, CD4 + T cells were costimulated for 2–3 days then infected in bulk by suspending 1.5 × 10^6^ cells in 250 μL of RF10 media containing 20 U/mL of IL-2, 10 μg/mL of polybrene, and 75 ng of HIV p24. Infections were allowed to proceed for 2 h on a rocking platform at 37 °C. Cells were then washed with media, and 10^5^ cells per well were seeded in 100 μL of RF10 containing the relevant concentration of compound. The infected cultures were incubated for 3 days, and then cell-free supernatants were harvested. Supernatants were diluted in PBS containing 2% Triton-x-100 and stored at 4 °C before quantification of HIV p24 protein levels by ELISA.

### 4.4 Cytokine quantifications

PBMCs were seeded at a concentration of 10^5^ cells per well in a v-bottomed 96 well plate, and then 200 μL of RF10 medium containing the relevant concentration of compound was added. After 18 h of incubation, the supernatant was removed from the cells and frozen at − 80 °C. Quantification of cytokines was performed using a custom Milliplex Human Cytokine Magnetic Kit (Millipore) according to the manufacturer's recommendations. Wash steps were performed using a Bio-Plex II Wash Station (Bio-Rad), and the data was acquired using a Bio-Plex 200 System with high-throughput fluidics (Bio-Rad). The resultant data was analyzed using Bio-Plex Manager 6.1 software.

### 4.5 Statistical analysis

For receptor and cytokine expression level comparisons two-sided *t*-tests were performed. A two-sided p-value < 0.05 between the groups was considered significant. Where cytokine concentration values were below the limit of detection, a value that was midway between 0 and the limit of detection was used. For synergy analysis, data were examined for outliers and contaminants to assess deviations from parametric model assumptions. Groups were then compared using exact one-sided Wilcoxon rank sum statistics, a nonparametric test. For PKC binding analysis the 95% confidence intervals were obtained from a one site competitive binding experiment using Graphpad Prism software where each concentration on the curve was run in triplicate.

### 4.6 J-Lat cell stimulations

J-Lat cells (clone 10.6) were incubated in 100 μL volume of RF10 medium containing the indicated concentrations of compound for 48 h before analysis. This was performed in v-bottomed 96-well tissue culture plates with a starting cell density of 25,000 cells/well. During harvesting, cells were washed and resuspended in 2% paraformaldehyde. The percentage of cells expressing GFP was then quantified by flow cytometry using a FC 500 flow cytometer (Beckman Coulter) and FlowJo software (version 7.6).

### 4.7 MTT assay

Cells were seeded in v-bottomed 96-well plates in a 200 μL/well volume of RF10 media containing the indicated concentration of compound. After 24 h cells were subjected to a 3-(4,5-dimethylthiazol-2-yl)−2,5-diphenyltetrazolium bromide assay (Vybrant MTT Cell Proliferation Assay Kit, ThermoFisher Scientific) according to manufacturer's instructions. Cells were incubated with substrate for 4 h, lysed and formazan solubilized, and then analyzed using a FLUOstar OPTIMA plate reader (BMG LABTECH).

### 4.8 PKC Binding assay

The PKC binding assay was performed as described previously (Marsden et al., 2017).
