# Supplementary material for: Characterization of designed, synthetically accessible bryostatin analog HIV latency reversing agents
Source: Virology. 2018 Jul;520:83–93. doi: 10.1016/j.virol.2018.05.006 (PMC6018613; doi:10.1016/j.virol.2018.05.006)
Supplement: Supplementary file 1 — Supplementary material [file mmc1.docx]

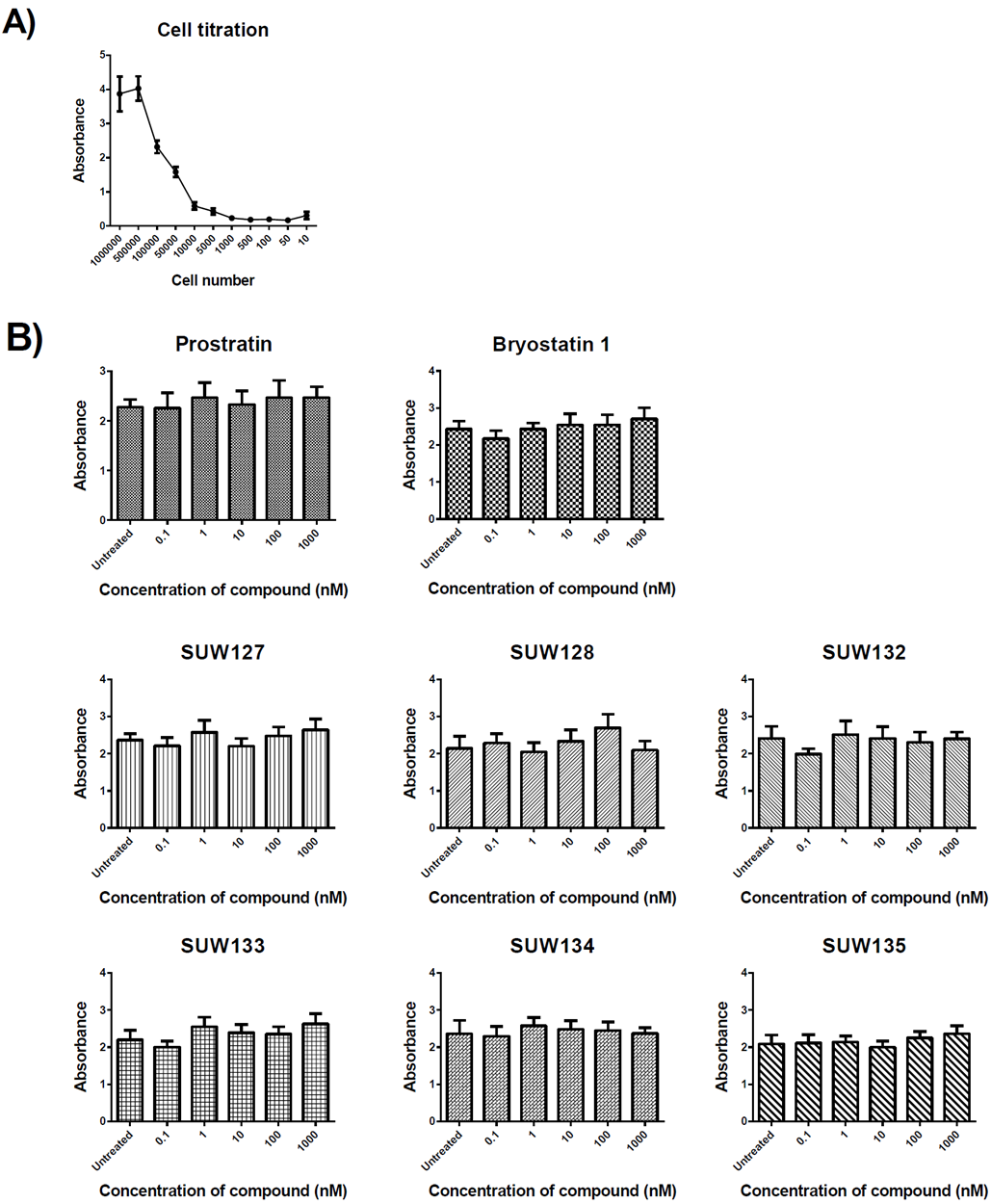


**Figure S1. MTT cytotoxicity assay in CEM T cells.** Cells were incubated for 24h and then assessed for viability using an MTT assay. **A)** Control cell titration without compound. **B)** 10,000 cells/well treated with the indicated concentration of compound. Error Bars = ±1SE (N = 4).

**
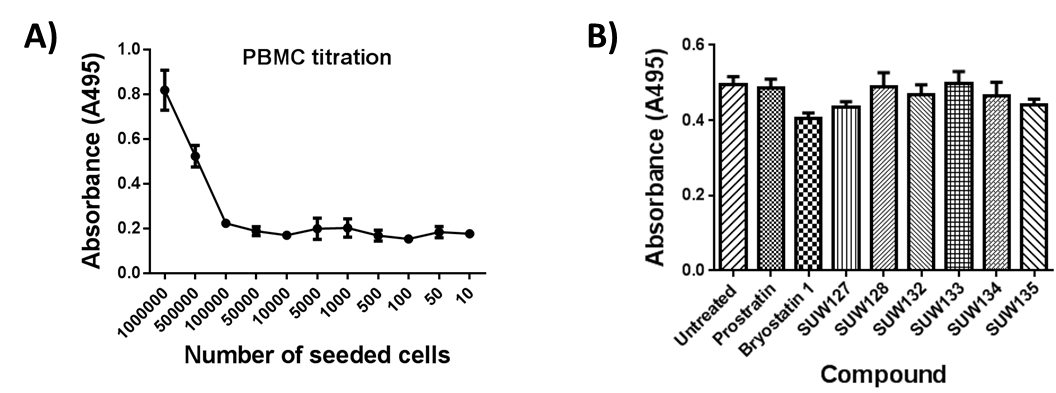
**

**Figure S2. MTT cytotoxicity assay in primary peripheral blood mononuclear cells.** Cells were incubated for 24h and then assessed for viability using an MTT assay. **A)** Control cell titration without compound. **B)** 500,000 cells/well treated with 100 nM concentration of compound. Error Bars = ±1SE (N = 4 donors and 12 replicate wells total).


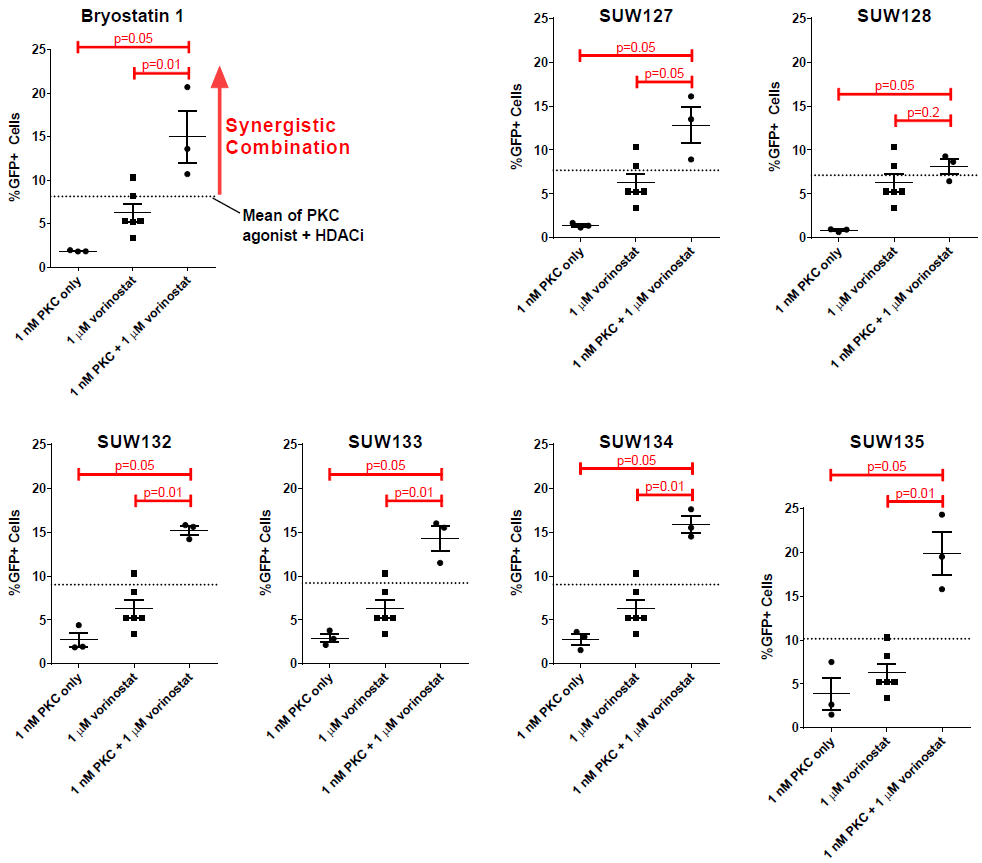


**Figure S3. Synergistic reactivation of HIV from latency.** J-Lat 10.6 cells were exposed to sub-optimal concentrations of the indicated PKC modulator alone (bryostatin 1 or SUW-designated compounds), the HDACi vorinostat alone, or a combination of the two for 48h. Lines representing the sum of PKC modulator alone and vorinostat alone, which is what would be expected from an additive response are shown for each PKC modulator. The same vorinostat alone values are shown in each plot to facilitate comparison. Groups were compared using an exact independent one-sided Wilcoxon rank sum test. P-values less than 0.05 are considered significant.


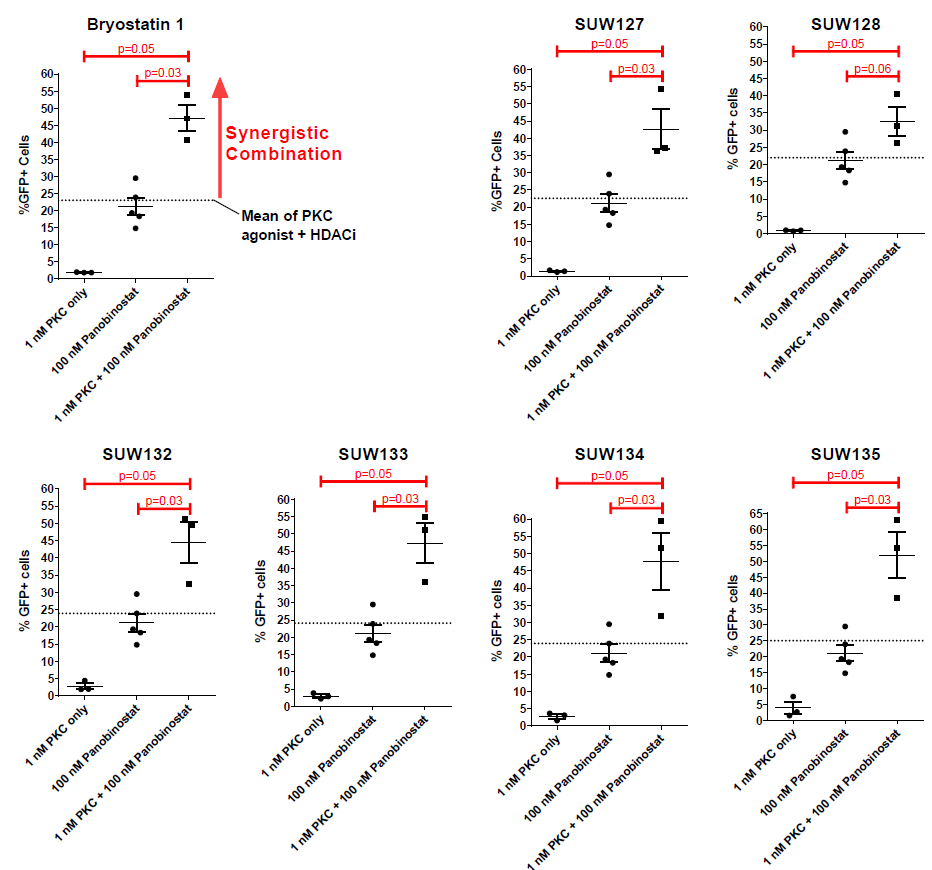


**Figure S4. Synergistic reactivation of HIV from latency.** J-Lat 10.6 cells were exposed to sub-optimal concentrations of the indicated PKC modulator alone (bryostatin 1 or SUW-designated compounds), the HDACi panobinostat alone, or a combination of the two for 48h. Lines representing the sum of PKC modulator alone and panobinostat alone, which is what would be expected from an additive response are shown for each PKC modulator. The same panobinostat alone values are shown in each plot to facilitate comparison. Groups were compared using an exact independent one-sided Wilcoxon rank sum test. P-values less than 0.05 are considered significant.
